# The shadow side of motives: self-destruction and dissociation in substance use disorders

**DOI:** 10.3389/fpsyt.2026.1780787

**Published:** 2026-03-24

**Authors:** Stéphane Potvin, Yasser Khazaal

**Affiliations:** 1 Centre de recherche de l’Institut Universitaire en Santé Mentale de Montréal, Montreal, QC, Canada; 2 Department of Psychiatry & Addiction, University of Montreal, Montreal, QC, Canada; 3 Addiction Medicine, Department of Psychiatry, Lausanne University Hospital, Lausanne, Switzerland

**Keywords:** dissociation, motives, self-destructive behavior, self-medication hypothesis, substance usage disorders

## 1 Introduction

Substance use disorders (SUDs) can be seen as incomprehensible or perhaps shocking to some people, as those affected with SUDs may persist in their consumption even though it is clearly associated with harmful consequences that are readily apparent to them and/or to those around them. To explain the phenomenon of SUDs, one of the main hypotheses that has been proposed is the self-medication hypothesis (1). This theory suggests that people struggling with SUDs, especially people with concomitant psychiatric disorders, are primarily motivated to consume psychoactive substances to relieve their symptoms (i.e. anxiety, depression, trauma-related distress and sleep problems (2). Accordingly, people often exhibit a psychopharmacological preference for specific substances (3). Empirically, this hypothesis is mainly supported by two observations: (i) many users report relief of anxious and depressive symptoms, or coping with negative emotions or stressful thoughts as one of their main reasons for using (4, 5); and (ii) some psychoactive substances (such as opioids) can indeed produce anxiolytic and antidepressant effects, at least in their initial acute phases (6). In addition, the high prevalence of concomitant psychiatric disorders among individuals with SUDs was seen as an epidemiological support consistent with the self-medication hypothesis (7, 8).

However, this hypothesis has been repeatedly criticized due to numerous pieces of evidence that do not align with its core assumptions (9, 10). First, self-medication is often not the main reason why people consume psychoactive substances as compared to other reasons such as the pursuit of positive enhancement (4, 11, 12) or social motives (13). If the self-medication hypothesis was completely accurate, one would expect, according to this hypothesis, to observe a clear match between the users’ symptoms and the substance they consume. For instance, individuals with anxiety would presumably prefer anxiolytic substances such as alcohol and people with attention deficit hyperactivity disorder (ADHD) would select only psychostimulants. Although this assumption is supported by some evidence (14, 15), there are many clinical situations where substance choice directly contradicts what would be expected based on the self-medication hypothesis. Classic examples include: (i) cannabis use among people with schizophrenia with negative symptoms (16, 17), (ii) the use of disinhibiting substances by people with cluster B personality disorders (18), (iii) cocaine use by patients with bipolar disorders during manic phases (19), and (iv) opioid use by people with ADHD (20). Moreover, substance choice is often influenced by other factors, such as availability and peer influence (21). Another piece of evidence contrary to the hypothesis lies in the fact that among individuals with SUDs and co-occurring psychiatric disorders, obvious problems of medication adherence have been documented (22). Non-adherence to prescribed treatments such as antipsychotics or antidepressants could be mentioned as an argument against the self-medication hypothesis. However, this may be explained by the delayed onset of therapeutic effects of medications compared to the possible transient immediate subjective relief provided by substances. Finally, further evidence against the self-medication hypothesis is that, beyond possible short-term relief from substances when used occasionally, long-term use is regularly associated with a worsening of psychiatric symptoms (16, 23).

## 2 Shadow motives

There is a world of difference between someone having a drink to relieve stress, and someone who consumes large quantities of substances to erase all thoughts or memories. While the self-medication hypothesis may explain substance use linked to coping motives (insofar as it describes the intentional use of substances to relieve specific psychological symptoms) and the pursuit of transient relief, it does not fully account for chronic, compulsive patterns, particularly in severe forms of SUD. Other motives, such as social and enhancement motives or other immediate gratifications motives, also contribute (5, 24). Yet, these motives still fall short of capturing more extreme cases—where individuals consume large quantities not to regulate discrete symptoms, but to detach from consciousness, numb unbearable emotions, or erase memories. These dissociative motives extend beyond coping and align with consistent evidence of episodic memory deficits in substance use disorders (25–28). Dissociative motives were missed from previous scales assessing motives for substance use (29, 30). Including dissociation (numbing, detachment, erasure of subjective experience) within existing motivational frameworks may help bridge the gap between symptom-focused explanations and the phenomenology of severe, compulsive use. This hypothesis seems coherent with the dissociative experiences that can be induced by several psychoactive substances, including alcohol (at high dose), opioids, cannabis and ketamine, as well as the self-reported desire to dissociate expressed by some people reporting drug use (31, 32).

Dissociative motives extend beyond symptom relief as described by the self-medication hypothesis to the pursuit of numbing, detachment, or the erasure of subjective experience. In this sense, they shift the focus from “using to feel better” toward “using to not feel at all” (33, 34). Such motives resonate more closely with a self-destruction hypothesis, insofar as they reflect a symbolic negation of the self and, in severe cases, a willingness to court harm through compulsive or extreme consumption. By highlighting this transition, dissociative motives help explain how patterns of use rooted in coping can evolve into profoundly destructive trajectories. It is fundamental to acknowledge, however, that dissociative motives are not necessarily self-destructive. In the short term, they may serve as a survival-oriented strategy to “switch off” unbearable emotions and seek temporary refuge in several situations (35). Yet, over time, the pursuit of detachment through heavy use can expose individuals to serious harm, blurring the boundary between a “survival strategy” and self-destruction. In severe SUDs, dissociation may thus shift to an expression of “self-destructive” drives (36). Yet, to avoid stigmatization, it is essential to describe behaviors as self-destructive rather than label individuals as being self-destructive. This raises the need to distinguish more clearly between dissociative motives and self-destructive ones. Dissociative motives are typically aimed at numbing, avoiding, or escaping intolerable states, with the implicit goal of preserving functioning in the face of overwhelming distress. Self-destructive motives, in contrast, involve a more direct orientation toward harm, where the behavior itself embodies or expresses an attack on the self. While these two motivational patterns may overlap or evolve into one another, differentiating them is critical for both clinical understanding and intervention.

Self-destructive behaviors are reported in individuals with SUDs in various forms such as self-injury (cutting, burning), suicide, and intentional substance overdosing (37, 38). In addition to these behaviors, other phenomena associated with SUDs may also belong to a dynamic of self-destruction. These include: (i) taking dangerous risks (e.g. driving under the influence), (ii) adopting unsafe lifestyle habits or unsafe modes of use increasing the risk for physical health problems (e.g. sexually or blood-transmitted infections, cardio- or cerebro-vascular diseases), and (iii) pursuing heavy substance use while cutting ties with work, home, school and/or family (39–42). Depending on the intent, these behaviors and choices may be motivated by a desire to hit “rock bottom”. Self-destructive tendencies are likely to manifest in a persistent manner and over extended periods, offering little respite to the individual. It is essential to understand these behaviors (and their motives), which differ from the self-medication hypothesis and from the transient pursuit of dissociation.

According to frameworks proposed to understand self-harm in borderline personality disorder, one may hypothesize that self-destructive behavior can emerge when an individual is confronted with a context or situation perceived as intolerable (43). Such behaviors may arise in large range of experiences such as dehumanization linked to perceived stigma and self-stigmatization, in moments of overwhelming shame, hopelessness or helplessness, or as an attempt to make visible a pain or anger that feels unheard and to provoke a reaction from significative others, or in reaction to complex trauma (44–47). These propositions resonate with the self-exclusion syndrome described among some people experiencing homelessness, where unbearable conditions lead to a radical severing of ties with self, others, and the broader social world (48). This concept highlights how extreme adversity, stigma, and hopelessness can drive not only social withdrawal but also self-destructive patterns, offering a lens to understand similar processes observed in addiction.

Finally, it is also conceivable that self-destructive phenomena may develop in an “autonomous manner”, without a specific social or psychological substrate, sustained instead by the severity of the addiction itself, the search for immediate rewards, and the difficulty of acting according to long-term consequences or regaining control over a behavior that has spun out of control (49). In such cases, however, the behaviors are self-destructive, but not the motives. Such views are coherent with contemporary functional neuroimaging research having shown that the functional connectivity between brain regions involved in decision-making and those involved in motivational impulses (e.g. reward seeking and punishment avoidance) is significantly disrupted in individuals with SUDs (50).

## 3 Discussion

Recognizing self-destructive behaviors, particularly when they persist over extended periods, is crucial. Distinguishing these clinical manifestations and identifying their underlying motives is essential for addressing such situations and providing appropriate support. A central challenge, however, lies probably in establishing contact and building a therapeutic alliance with the individual. Future studies may help to characterize such behaviors and assess potential interventions such as assertive community services and peer support (51, 52). When possible, psychotherapy including compassion-focused therapy approaches and self-compassion may be helpful to counteract the self-destructive processes (53, 54).

Future research should include people with lived experiences to assess the view presented in this opinion paper, which proposes that self-destructive behaviors may constitute important indicators of the severity of SUDs that should not be overlooked (55). Our goal is to encourage the acquisition of knowledge on this topic and to improve therapeutic response to such clinical phenomena.
